# Overlay or Overlie

**Published:** 1908-05-30

**Authors:** 


### OVERLAY OR OVERLIE?

When Pascal crystallised into a sentence the first two rules which govern the science of logic, he laid down once for all the foundation of man's efforts to solve the problems of his own existence and of the universe. "To define all terms, and to prove all propositions," is indeed a task which in everyday life might prove rather troublesome, but it is the basis, if occasionally unrecognised, of all scientific method and research. Professor Sir Clifford Allbutt, too, has well impressed upon his students that what symbols are to the mathematician, words should be to the scientist; that is, we take it, the expressions each of a perfectly definite conception of the mind such as can be conveyed in no other way. Indeed, there are precisians who tell us that no true synonym exists in the language, but that there are subtle shades of distinction which do convey a difference between even the nearest approximations in significance. The scientist is debarred from ever offering proofs of his conclusions as "rigid" as the demonstrations of the mathematician, but that does not exonerate him from the burden of most carefully selecting the words he uses, and of making sure that both he himself and his audience have clear perceptions of the exact value of these symbols. He must, mentally at least, be able to define his terms.

An interesting example of the necessity for strict adherence to these principles arose the other day in Parliament during the consideration in committee of the Childrens Bill. A sentence in Clause 14 ran thus: "If any person causes the death of an infant by overlying it," and this was amended by the substitution of "overlaying" for "overlying." The point was submitted to Dr. J. A. H. Murray, as the highest available authority on the terminology of the English language, who decided in favour of the proposed alteration. At first sight one might feel inclined to argue that since "to lie" is intransitive, whereas "to lay" is the transitive form, the same should hold good about the compound verbs; but it seems that the matter is hardly so simple as might be supposed. After an illuminating discussion of the pros and cons, Dr. Murray finally sums up in favour of "to overlay," past tense and past participle "overlaid"; this, too, is the verb adopted in the Authorised Version of 1611, though Wiclif before that had used the other.

The ease with which a coach and four can be driven by an expert lawyer through any Act of Parliament is proverbial, but it is perhaps a sign of better times that the Legislature should be so scrupulously accurate and critical in details of this sort. For in point of fact even scientists, and, worse still, even logicians, are often guilty of gross and careless misuse of the words with which, as symbols of their mental concepts, they seek to establish a conclusion or elaborate an argument; and our own science of medicine can furnish, perhaps, as many instances as any other of this laxity of thought and habit. "Case" is a word which all but the most careful do violence to in unwary moments. One may treat, if one is fortunate, so many scores or hundreds of cases of influenza, let us say; but it is incorrect to write, as there is great temptation to do, that some other number of these cases died or recovered. The patient, whose symptoms of disease and the course they run constitute a "case," can die or recover; but the purely abstract notion to which alone the term "case" is applicable can, of course, do nothing of the sort. Yet everyone who has contributed to the vast mass of medical literature has probably fallen occasionally into this very common error; and even those who have the valuable faculty of being able to revise their own products with a critical eye do not always escape the pitfall. Of similar instances the list could be almost indefinitely extended. Nor is such ultra-carefulness to be lightly put aside as mere pedantry. Precise thought can only find expression in precise writing, and without precise thought we cannot expect to reason with any approach to correctness.

